# PSYCHOLOGICAL WELL-BEING AND PHYSICAL HEALTH IN THE CONTEXT OF OUR RAPIDLY AGING WORLD

**DOI:** 10.1093/geroni/igad104.0534

**Published:** 2023-12-21

**Authors:** Eric Kim

**Affiliations:** University of British Columbia, Vancouver, British Columbia, Canada

## Abstract

Our society is rapidly aging. Thus, citizens, researchers, and policymakers alike are seeking ways to re-engineer society to achieve the dual aim of helping our aging population age well and fostering new ways for older adults to deploy the abundance of strengths that accrue with age. Most public health, biomedical, and psychological efforts have focused on reducing harmful risk factors. This approach has contributed greatly to prevention and treatment programs. However, another approach to achieving this dual aim is to expand the focus and evaluate upstream dimensions of psychosocial well-being. This approach might help inform the multidisciplinary and multi-level response efforts we need. In this talk, I will describe a theoretical model, results from a series of studies evaluating associations between a sense of purpose with reduced risk of chronic conditions, and mechanistic biobehavioral processes underlying these associations. This work aims to provide new directions for building a science of resilience and providing new targets for multi-level interventions that we can weave into our daily lives via practices and systems.

